# A Case in Prof. Moore’s Clinic at the Buffalo Hospital, Sisters of Charity; Resection of the Elbow

**Published:** 1863-01

**Authors:** 


ART. IV.—*A Case in Prof. Moore's Clinic at the Buffalo Hospital, Sisters of Charity; Resection of the Elbow.*

Catharine Haynes, married, aged 34, her youngest child 4 years old, began to have pain in right elbow joint on 5th September, 1860; continued to use her arm for two months; pain all the while growing gradually worse, and swelling being developed; swelling very palpable in March, 1861; was opened by Dr. Wilcox in July following for abscess of joint; from this time it continued to discharge till operated upon before the class November 19, 1862.

The operation was commenced by exploration, in order to detect the exact seat of caries; the line of cut being nearly parallel with the axis of the arm, commencing at the cloaca, and extending over the external condyle for about two inches in length at first. The finger introduced revealed the fact that caries existed on both articular surfaces; the cut was now elongated to about four inches; another parallel incision was made over the internal condyle, having its centre opposite the joint. The two were now connected by a transverse incision, dividing the triceps muscle at its junction with the olecranon, thus performing the operation in "H." The arm being now forcibly flexed, the olecranon was removed by a saw. The ulnar nerve was carefully dissected from its bed, guarded by the finger nail. Dissection of soft parts for a short distance above the articular surface enabled a narrow blade, with the cutting edge reversed, to be pushed to the anterior surface of humerus. The removal of the whole articular surface was effected by sawing from before backward. The head of radius was removed in a similar manner. But one arterial branch required ligation. The part was now carefully sponged, and interrupted sutures introduced, leaving an opening along the internal cut for the exit of blood or pus; adhesive straps with cold water dressings completed the operation. The patient was placed in bed, with her arm supined and laid on a pillow.-- Two days afterward (November 21st,) no constitutional disturbance whatever--no hemorrhage or other discharge. On 26th still no discharge, apparently union by adhesion over the whole surface. On the 27th slight purulent discharge, (stitches had been removed on the fourth day.) Pus continued to discharge till December 14th. On this day an erysipelatous inflammation appeared in the arm, which was treated by a local application of tinc. ferri chloridi, a cathartic of sulph. magnesia was also administered. December 19th erysipelatous blush almost disappeared. A slightly angular splint, extending from the hand to the shoulder was applied for the purpose of bringing the arm from the extended position in which it was placed after the operation, up to a rectangular position, in order to prevent anchylosis, or failing in this to secure a more useful attitude.

